# Standard versus prosocial online support groups for distressed breast cancer survivors: a randomized controlled trial

**DOI:** 10.1186/1471-2407-11-379

**Published:** 2011-08-25

**Authors:** Stephen J Lepore, Joanne S Buzaglo, Morton A Lieberman, Mitch Golant, Adam Davey

**Affiliations:** 1 Temple University, Department of Public Health, 1301 Cecil B. Moore Ave., 9th Floor Ritter Annex, Philadelphia, PA, 19122, USA; 2 Cancer Support Community, Research & Training Institute, 4100 Chamounix Drive, Philadelphia, PA, 19131, USA; 3 University of California San Francisco, 1104 Western Ave, Mill Valley, Ca. 94941, USA

## Abstract

**Background:**

The Internet can increase access to psychosocial care for breast cancer survivors through online support groups. This study will test a novel prosocial online group that emphasizes both opportunities for getting and giving help. Based on the helper therapy principle, it is hypothesized that the addition of structured helping opportunities and coaching on how to help others online will increase the psychological benefits of a standard online group.

**Methods/Design:**

A two-armed randomized controlled trial with pretest and posttest. Non-metastatic breast cancer survivors with elevated psychological distress will be randomized to either a standard facilitated online group or to a prosocial facilitated online group, which combines online exchanges of support with structured helping opportunities (blogging, breast cancer outreach) and coaching on how best to give support to others. Validated and reliable measures will be administered to women approximately one month before and after the interventions. Self-esteem, positive affect, and sense of belonging will be tested as potential mediators of the primary outcomes of depressive/anxious symptoms and sense of purpose in life.

**Discussion:**

This study will test an innovative approach to maximizing the psychological benefits of cancer online support groups. The theory-based prosocial online support group intervention model is sustainable, because it can be implemented by private non-profit or other organizations, such as cancer centers, which mostly offer face-to-face support groups with limited patient reach.

**Trial Registration:**

ClinicalTrials.gov: NCT01396174

## Background

Ongoing and timely access to relevant information and social support can improve psychological outcomes in breast cancer patients [1], but such access is far from universal [2]. The Internet can increase access to psychosocial care through online support groups (OSGs), yet the few randomized trials of cancer OSGs have yielded mixed results [3-6]. Thus, there is a need to develop innovative approaches to maximizing the psychological benefits of OSGs. An obvious benefit of a support group is access to information and emotional support from similar others. Less obvious are the benefits accrued from providing help to others. According to the "helper therapy principle," [7] helping benefits the helper, possibly enhancing psychological and physical health outcomes [8-11]. Based on this theory and prior research, we hypothesized that OSGs that maximize opportunities for providing help to others would be highly efficacious at reducing distress symptoms.

Depression and anxiety symptoms are highly prevalent among breast cancer survivors [12] and often undertreated [13]. A systematic review found high rates of probable depression in breast cancer patients (10% to 25%) [13]. In a rare 5-year cohort study, nearly 50% of women with early breast cancer had depression, anxiety, or both one year after diagnosis, 25% in the second, third, and fourth years, and 15% in the fifth year [14]. Controlling distress is important in its own right, but clinically it may be important to address psychological distress in breast cancer patients in the early years after treatment because it can be detrimental to quality of life, treatment adherence, cancer surveillance, health behaviors (e.g., exercise, diet), self-care (e.g., managing lymphedema), and overall health outcomes including risk for recurrence, and decreased immune function [12,15-19]. Breast cancer may bring about distress, in part, through its adverse effects on self-esteem [20], sense of social belonging [21], and sense of purpose [22,23]. Amount of helping others has been shown to be positively related to all of these outcomes and inversely related to depression [24,25]. Thus, an intervention promoting prosocial behavior might reduce distress in breast cancer survivors directly and indirectly through these psychological correlates of distress.

Dozens of interventions have been designed to reduce distress in cancer survivors, but the evidence on their efficacy and acceptability is weak [26,27]. Moreover, many interventions that require in-person contact are expensive and do not reach patients who might be constrained by time, money, mobility, or location. OSGs are a fairly low cost alternative to in-person interventions and overcome many of the obstacles of in-person interventions. Breast cancer survivors often use OSGs [28], so this population should be receptive to an OSG intervention.

To date, the evidence on the efficacy of cancer OSGs is limited. Winzelberg et al. [3] randomized 72 breast survivors to a professionally facilitated OSG or a wait-list control group. Compared with the controls, OSG participants had significantly greater reductions in depressive symptoms (effect size = 0.54) from baseline to follow-up. Fifty-three percent of OSG participants who were depressed at baseline were below the clinical cut-point at follow-up, compared with only 29% improvement in the control group. Owen et al. [6] randomized 62 breast cancer survivors to an Internet-based coping-skills training group (with access to a non-facilitated peer discussion board) or to a wail-list control group and found no intervention effects on quality of life. Similarly, Salzer et al. [4] randomized 78 breast cancer survivors to a non-facilitated OSG or to an Internet-based education control group and found no intervention effects on distress or quality of life. And, finally, Gustafson et al. [5] randomized 295 breast cancer survivors to a non-facilitated OSG or to a control group and found no intervention effects on quality of life. Given this mixed evidence, we cannot draw firm conclusions on the efficacy of cancer OSGs. The field must identify ways to maximize the psychological benefits derived from OSGs if they are to be recommended.

The current study will develop and evaluate a novel OSG intervention that theoretically should boost the benefits observed in standard facilitated OSGs [3,29]. Most social support research focuses on the health benefits of *receiving *social support [30], but there is evidence that *providing *social support also confers benefits [11,31-33]. Some research even suggests that providing support is more beneficial than receiving support [10,31]. In general, engagement in prosocial actions (e.g., helping, advising) appears to enhance health and quality of life outcomes [33,34]. However, the bulk of empirical evidence on the benefits of helping comes from observational studies with non-clinical populations. Studies using controlled experimental [35] or longitudinal observational designs [36] are rare. The current trial is the first to evaluate the psychological benefits of increasing helping behaviors among distressed cancer survivors in the context of an online supportive intervention.

**Figure 1:**
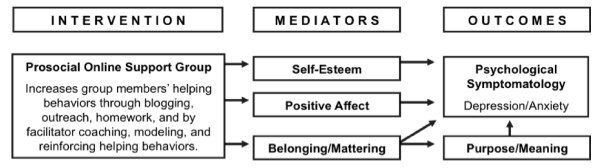
**Conceptual model for Life Beyond Cancer trial**.

The trial also aims to identify potential mechanisms underlying the psychological benefits of helping. Based on the literature and Midlarsky's [33] conceptual framework, we developed the model in Figure 1. The model identifies several potential mediating variables that have been linked both to helping behavior and to psychological distress. Helping has been positively correlated with self-esteem [37] and positive affect [38], which, in turn, have been negatively correlated with psychological distress [39,40]. The role of positive affect is of particular interest given the evidence that positive emotions do not just signal optimal psychological functioning, but they can produce it [41]. Positive emotions also can facilitate coping with stress [42]. Helping others also can increase a sense of belonging, or mattering to others [43], which, in turn, has been linked to lower levels of depression and anxiety [44,45]. Further, we anticipate that sense of belonging will be indirectly related to psychological distress through its effects on sense of purpose. The formation of interpersonal attachments has been called a fundamental human need [46]. Belonging to a group and having a role in that group can have a powerful influence on one's sense of purpose [47]. Cancer survivors often report a diminished sense of purpose and meaning in life [22,48]. In turn, a lack of purpose is associated with psychological symptoms of depression and anxiety [49]. If establishing meaningful connections with others can promote or restore a sense of purpose, it could also reduce distress [50].

### Objectives and Hypotheses

The major objective of the "Life Beyond Cancer" trial is to test the efficacy, acceptability, and feasibility of a novel OSG for distressed breast cancer survivors that provides structured helping opportunities and coaching on how best to give support to others. The trial targets distressed breast cancer survivors because they will likely benefit more than non-distressed patients from psychosocial interventions [51]. The study will explore whether the enhanced OSG increases the exchange of helping behaviors among distressed breast cancer survivors and, consequently, reduces their psychological distress symptoms and increases their sense of purpose relative to their counterparts in a standard OSG. Secondarily, we will evaluate potential psychological mediators of the enhanced, prosocial OSG, and evaluate the acceptability and feasibility of implementing the prosocial OSG among distressed breast cancer survivors.

Hypothesis 1: Relative to women in the standard OSG (S-OSG) condition, women in the prosocial OSG (P-OSG) condition will have a lower mean level of depression/anxiety symptoms at posttest.

Hypothesis 2: Relative to women in the S-OSG condition, women in the P-OSG condition will have a significantly greater mean level of sense of purpose in life, self-esteem, positive affect, and sense of belonging at posttest.

Hypothesis 3: Positive increases (posttest-pretest) in mean level of self-esteem, positive affect, and sense of belonging will partially mediate the effects of the P-OSG intervention on mean level of depression/anxiety symptoms and sense of purpose at posttest.

Hypothesis 4: Positive increases (posttest-pretest) in mean level of sense of purpose will partially mediate the effects of the P-OSG intervention on mean level of depression/anxiety symptoms at posttest.

Hypothesis 5: Given the equal level of attention across conditions, we hypothesize that there will be no significant between group differences in retention (percent completing study) and mean level of satisfaction with the intervention.

## Methods/Design

A two-group randomized controlled trial design with pretest and posttest measures will be used to address the study aims. Distressed breast cancer survivors will be randomized equally to one of two groups: (a) P-OSG, a facilitated OSG with structured helping opportunities and coaching on how to recognize and be responsive to others needs for help or (b) S-OSG, a comparison standard facilitated OSG. Data will be collected approximately one month before and after the intervention. The study design is guided by the CONSORT criteria [52]. The study is in compliance with the Helsinki Declaration and has received approval from the Temple University Institutional Review Board (protocol number 13705).

### Participants

Eligibility inclusion criteria are: diagnosed with stage I or II breast cancer within the preceding 36 months; age 21 to 65 years (the age cap minimizes sampling women with cognitive or physical impairments that could limit participation and maximizes sampling women with computer experience), access to and ability to use a computer and the Internet, able to understand the informed consent form, fluent in spoken English, and meet the screening criteria for psychological distress. Women will qualify as distressed if they fall above the "non-case" cut-point (≥ 8) on either the depression or anxiety subscale of the Hospital Anxiety and Depression Scales (HADS [53]). This approach will generate a sample with a range of subclinical and clinical levels of distress symptoms [54]. We will not exclude women who previously have participated in an OSG or received other psychosocial treatments. Instead we will measure these variables and include them as covariates in analyses if necessary.

### Procedures

The sampling frame will be generated in conjunction with a State Cancer Tumor Registry and will target women meeting the age and diagnostic criteria. Advance letters will be sent to potential participants. Respondents will be screened for eligibility using standardized measures administered over the phone. Eligible participants will be consented. Non-eligible participants will be sent a resource list.

Trained research assistants will collect data via structured telephone interviews. A random subset of interviews will be monitored and reviewed for quality control. Interviewers will be blind to participants' intervention condition. Within one month following end of treatment, participants will be re-interviewed to collect follow-up data. Again, a random subset of interviews will be reviewed for quality control and interviewers will remain blind to participants' condition.

Because age is related to level of Internet use [55] and to level of psychological distress in breast cancer samples [56], age-stratified block randomization (< 51 vs. 51+ years) will be used to assign women to condition and ensure that age is balanced across treatment arms. The project statistician (AD) generated the random sequence using a random numbers generator within STATA (STATACorp, LP, College Station, TX, 2011). Following baseline data collection, the project director, who will be blind to participants' baseline data, will determine participants' assignment using the randomization schedule and inform the group facilitator via email. The facilitator will contact the participant with further instructions on group participation.

Facilitators will be graduate-level, trained health professionals. Trained researchers will use treatment fidelity checklists to evaluate facilitators' adherence to study protocols. Co-investigators will conduct supervision sessions with facilitators to review treatment fidelity and to address any problems.

### Measures

All major study measures have established reliability and validity. The primary outcome measure will be the HADS, which has been used extensively and validated with breast cancer and other cancer patient populations [57]. The HADS can be used to generate continuous measures of symptoms of depression and anxiety and also provides clinical cut-offs. The secondary outcome, sense of purpose, will be measured using the validated and reliable Meaning in Life Questionnaire [58]. Validated and reliable measures also will be used to assess the three mediators: self-esteem with Rosenberg's Self-Esteem scale [59], positive affect with the Positive Affect Scale [60], and sense of belonging (i.e., mattering to others) with the General Mattering Scale [61].

Measures of potential covariates include background variables (e.g., age, race, treatments) and other potential confounding variables [e.g., Social Provisions Scale [62] to measure social support received by social network members, Comorbidity Questionnaire [63] to measure comorbid health conditions, and questions about receipt of formal (e.g., counseling, medication) and informal (e.g., support groups) psychosocial interventions including participation in online support groups unrelated to the study]. Level of "satisfaction" with and perceived "helpfulness" of different intervention elements (e.g., support received, opportunities to help others, interactions with facilitators) will be assessed using 5-point Likert-type scales (1 = very unsatisfied to 5 = very satisfied).

Finally, as a manipulation check, we will assess if the P-OSG intervention increased helping behaviors relative to the S-OSG. Research assistants will code instances of online support provisions using Bambina's Support OnLine (SOL) coding scheme [64]. SOL can be used to reliably code major categories of informational and emotional support provision. Coders will be blind to intervention condition. As part of the process assessment, P-OSG participants also will answer questions about perceived barriers or concerns with the "outreach" and "blogging" activities (see below).

### Interventions

#### Standard online support group intervention

The S-OSG comparison condition is based on an empirically validated facilitated breast cancer OSG [3,29]. The intervention is manualized and interventionists will receive regular monitoring and supervision by PhD-level clinical psychologists with experience running cancer OSGs. The OSGs are modeled after face-to-face support groups [e.g., [65,66], such as those conducted in community settings throughout North America (i.e., Gilda's Club, Cancer Support Community). While the proposed OSG is distinct in that specific topics are introduced at each session, several components remain consistent with the face-to-face model; including all sessions are professionally led and participants are considered the experts in their own experience. The groups will meet online weekly for approximately 1.5 hours and participants will be able to email and chat. Each week the facilitator will introduce a new topic and encourage participants to share their relevant experiences, concerns, and problems. The facilitator will not encourage people to help others, but instead focus on promoting self-expression (e.g., "Tell us more about the pain in your arm.").

The weekly topics, which were drawn from research on breast cancer survivorship [e.g., [67-69], include: (1) symptoms of pain, fatigue and lymphedema; (2) self-esteem and body image concerns; (3) problems in physical and recreational activities; (4) problems in intimacy, sexual interest and function; (5) coping with depression, anxiety, and fears of recurrence; and (6) challenges to staying healthy (diet, exercise, surveillance). In addition, as a control for the "helping blog" activity in the P-OSG condition (see below), participants in the S-OSG will be asked to select one of the six weekly topics that they wish to write about as a way to organize some of their thoughts and feelings. This type of writing is similar to expressive writing, which no study to date has found to be effective in reducing psychological distress in breast cancer survivors [70], so it serves as an effective attention control for the "helping blog" activity. The writing will be private and not shared with anyone, including the research team.

#### Prosocial online support group intervention

The P-OSG intervention includes elements of the S-OSG, but with critical modifications and key additions. First, participants will receive a tip sheet that describes how to recognize others' needs for different kinds of support and how to be helpful in an online environment. Second, the facilitator will post reminders at the beginning of each session on the importance of responding to one another's needs for help. Third, participants will be asked to commit to a goal of offering informational or emotional support to group members each week during the live synchronous sessions or later using the discussion board, email or chat. Fourth, prior to each week's meeting, participants will receive an email describing the topical problem to be discussed (e.g., pain, fatigue, and lymphedema). Their "homework" is to prepare 1-2 sentences on how their experiences with the problem might help others to cope with the problem. If they have not had the problem, they will be asked to write 1-2 sentences on how their approach to coping with other life problems might be useful to group members coping with the particular problem of the week. Fifth, the facilitator will encourage helping by highlighting direct and indirect requests for support by group members, will praise those who offer help (e.g., "Nice suggestion, Margie."), and will encourage additional help (e.g., "Has anyone else found a good way to cope with the problem Joan mentioned?").

Members of the P-OSG also will be asked to select one of the six weekly topics to prepare a blog about the topic to share with the group. The goal will be to write about their experience as a way of helping other breast cancer survivors who have gone through a similar experience. The writing instructions will emphasize that participants should not worry about grammar or spelling and that if they wish the research staff can help to edit their writing for grammar or spelling before posting. Participants will be asked to blog about any effective ways they have developed to cope with a cancer-related problem. The exercise will emphasize that people often have experiential knowledge that is valuable to others, but can only be accessed if there is sharing. Bloggers will be told that the exercise is designed to help other patients to understand the different consequences of breast cancer and its treatment, as well as how they might best manage or address these consequences. Instructions also will explain that the bloggers do not necessarily have to provide answers or solutions to the problems they write about, they simply need to share their experience. Reading others similar experiences can help to normalize the experience or make readers feel less alone. We recognize that some participants might be resistant to sharing their writings with others, either because they do not feel it will be valuable or they feel it is too personal. We will offer individuals the opportunity to have their blog posted anonymously, or only post select information, or for us to paraphrase what they have written in a list of comments comprised of input from multiple blogs. All blogs will be fact checked by research staff to avoid posting misinformation.

Finally, in addition to promoting helping within the group, the P-OSG will promote helping outside of the group through an outreach activity called the "Breast Cancer Awareness Ambassadors" program. Women in the P-OSG group will be asked to increase breast cancer awareness by sharing a "Breast Cancer and You" fact sheet and "Mammograms Save Lives" e-Cards, both developed by the Centers for Disease Control and Prevention, with women in their social network who are 40+ years old.

### Analytic Plan

Descriptive statistics will be generated for the total sample and the treatment groups for each time point. Distributional properties will be analyzed to determine if variance stabilizing or normalizing transformations should be applied. Outliers will be assessed and checked for accuracy. Non-response and attrition must be addressed in longitudinal research [71]. Therefore, full information maximum likelihood (FIML) and multiple imputations (MI) will be used to correct for missing data [71]. Variables associated with missing data mechanisms will be included as covariates in models or used to create multiple imputations as appropriate. Both FIML and MI are implemented in a variety of software packages including LISREL, AMOS, and MPlus, each of which can accommodate all statistical models (ANCOVA, OLS and logistic regression, mediation models, etc.) that will be estimated in this project.

Hypotheses 1 and 2: Post-test differences between P-OSG and S-OSG in primary and secondary outcomes will be tested using ANCOVA, with intervention group as the predictor and appropriate pre-test scores as covariates. Intent-to-treat analyses will be used. Variance associated with cohort membership will be estimated via intraclass correlation coefficients. If necessary cohort can be entered into the ANCOVA models as a random effect or fixed-effects analyses can be performed if there is evidence for unobserved heterogeneity. Group-level analyses can be performed on aggregate data if necessary.

Hypotheses 3 and 4: Mediation will be tested in a series of ANCOVA models using standard statistical software or structural equation modeling software. Hypothesis tests will be based on bootstrapped standard errors [72].

Hypothesis 5: Acceptability will be assessed by recruitment and retention rates, reasons for refusal/dropout, and level of participants' ratings of satisfaction and helpfulness of different intervention elements. Fisher's exact test will be used to evaluate differences between conditions for categorical outcomes; t-tests or non-parametric equivalents can be performed to compare ordinal or continuous outcomes.

The feasibility of manipulating online helping behavior will be assessed by comparing mean levels of observed helping behaviors (using SOL data) enacted by participants in the P-OSG versus S-OSG and participants' willingness and ability to generate a "helping blog" and engage in "outreach" activities in the P-OSG condition. Additional analyses will be conducted to confirm that treatment fidelity was equivalent across conditions and to explore dose-response relations by examining whether level of participation (e.g., attendance, number of posts, amount of help provided to others) moderates intervention effects.

Because this is a preliminary study of the effect of the novel P-OSG intervention, we are interested in estimating effect sizes of the P-OSG intervention group. More specifically, we are interested in gaining an estimate of the true treatment effect size (which is independent of sample size) and to establish a plausible confidence interval around this effect size. Thus, we present information regarding the aim of accuracy in parameter estimation (AIPE) [73] as well as information relevant to the aim of null hypothesis significance testing.

AIPE identifies a range (half-width, *w*) within which an effect size can be bracketed with a specified probability, independent of the magnitude of the observed effect size. It is easiest to interpret in the metric of standardized regression coefficients, i.e., β ± *w*. For these analyses, AIPE will depend on (a) the number of predictors, (b) variance in predictor of interest accounted for by other predictors, $R_{xx}^{2}$, and (c) overall variance predicted in outcome, $R_{yx}^{2}$. Our models will have between 2 (pre-test & treatment) and 10 predictors (i.e., adding 3 mediators and possibly 5 cohort identifier dummy variables). We estimated half-widths assuming: model $R_{yx}^{2}$ was either high (.5) or low (.1) and $R_{xx}^{2}$ was zero (treatment), low (.1) or high (.5). With 80% probability for N = 180 (90 per experimental condition), we expect to be able to bracket effect sizes for treatment effects within ± .11 to ± .15 depending on model R^2^. Effect sizes for mediators will be estimated with somewhat less precision but within ± .12 to ± .22

Power to identify post-test differences between P-OSG and S-OSG controlling for pre-test values and group membership depends on the correlation between pre-test and post-test scores. Minimum detectable effect size $\left(\hat{d}\right)$ with power = .8 and α = .05, is well approximated (R^2 ^> .99) for specific pre-post correlations (r_01_) by the formula: $\hat{d}=0.41+0.08r_{01}-0.36r_{01}^{2}$ and ranges from .19 (r_01 _= .9) to .42 (r_01 _= 0).

## Discussion

In terms of clinical practice, we believe that the current lack of conclusive evidence on the efficacy of OSGs has stymied the broader implementation and dissemination of facilitated OSGs. The majority of National Cancer Institute-designated cancer centers in the United States, as well as community-based hospitals and cancer centers, primarily offer face-to-face supportive care, which is costly and has limited patient reach. If successful, results of the proposed trial could stimulate broader implementation of facilitated OSGs and offer an innovative approach to implementing OSGs that emphasizes prosocial behavior within the group and outside the group. Specifically, the proposed prosocial OSG model offers an alternative to the dominant self-help OSG, which lacks facilitation and emphasizes emotional expression over mutual exchange of help. Importantly, the prosocial OSG provides a model for practice that can be easily adopted by organizations that offer psychosocial care to cancer survivors.

## List of abbreviations

The following abbreviations were used: AIPE: accuracy in parameter estimation; ANCOVA: Analysis of covariance; FIML: full information maximum likelihood; MI: multiple imputation; OLS: ordinary least squares; OSG: 0nline support group; P-OSG: prosocial online support group; S-OSG: standard online support group.

## Competing interests

The authors declare that they have no competing interests.

## Authors' contributions

SJL, JB, ML, MG developed the study concept and aims. AD assisted with the analytic plan. SJL drafted the manuscript. SL and JB will implement the protocol. SL will oversee enrollment and data collection. JB, ML and MG will oversee facilitator training and monitoring. AD will assist with sampling, randomization procedures and data analysis. All authors contributed to and approved the final manuscript.

## Pre-publication history

The pre-publication history for this paper can be accessed here:

http://www.biomedcentral.com/1471-2407/11/379/prepub
